# Trace Elements as Immunoregulators in SARS-CoV-2 and Other Viral Infections

**DOI:** 10.1007/s12291-021-00961-6

**Published:** 2021-02-12

**Authors:** Karthick Dharmalingam, Amandeep Birdi, Sojit Tomo, Karli Sreenivasulu, Jaykaran Charan, Dharmveer Yadav, Purvi Purohit, Praveen Sharma

**Affiliations:** 1 Department of Biochemistry, All India Institute of Medical Sciences, Jodhpur, Rajasthan India; 2 Department of Pharmacology, All India Institute of Medical Sciences, Jodhpur, Rajasthan India

**Keywords:** Trace elements, Cell-mediated immunity, Zinc, Selenium, Copper, Manganese, Cytokines, Reactive oxygen species, COVID-19

## Abstract

Nutritional deficiency is associated with impaired immunity and increased susceptibility to infections. The complex interactions of trace elements with the macromolecules trigger the effective immune response against the viral diseases. The outcome of various viral infections along with susceptibility is affected by trace elements such as zinc, selenium, iron, copper, etc. due to their immuno-modulatory effects. Available electronic databases have been comprehensively searched for articles published with full text available and with the key words “Trace elements”, “COVID-19”, “Viral Infections” and “Immune Response” (i.e. separately Zn, Se, Fe, Cu, Mn, Mo, Cr, Li, Ni, Co) appearing in the title and abstract. On the basis of available articles we have explored the role of trace elements in viral infections with special reference to COVID-19 and their interactions with the immune system. Zinc, selenium and other trace elements are vital to triggerT_H_1 cells and cytokine-mediated immune response for substantial production of proinflammatory cytokines. The antiviral activity of some trace elements is attributed to their inhibitory effect on viral entry, replication and other downstream processes. Trace elements having antioxidants activity not only regulate host immune responses, but also modify the viral genome. Adequate dietary intake of trace elements is essential for activation, development, differentiation and numerous functions.

## Introduction

Human being have witnessed three major epidemics caused by coronaviruses such as Severe Acute Respiratory Syndrome (SARS-2003), Middle East Respiratory Syndrome (MERS-2012) and novel Coronavirus (n-COV) Disease throughout the world in the recent past [1, 2]. COVID-19 was emerged in Wuhan city, China in December 2019. On 12th January 2020, World Health Organization (WHO) named it as novel coronavirus, and later on 11th February 2020 it was renamed as Severe Acute Respiratory Syndrome Coronavirus-2 (SARS-CoV-2) by WHO.

**Fig. 1:**
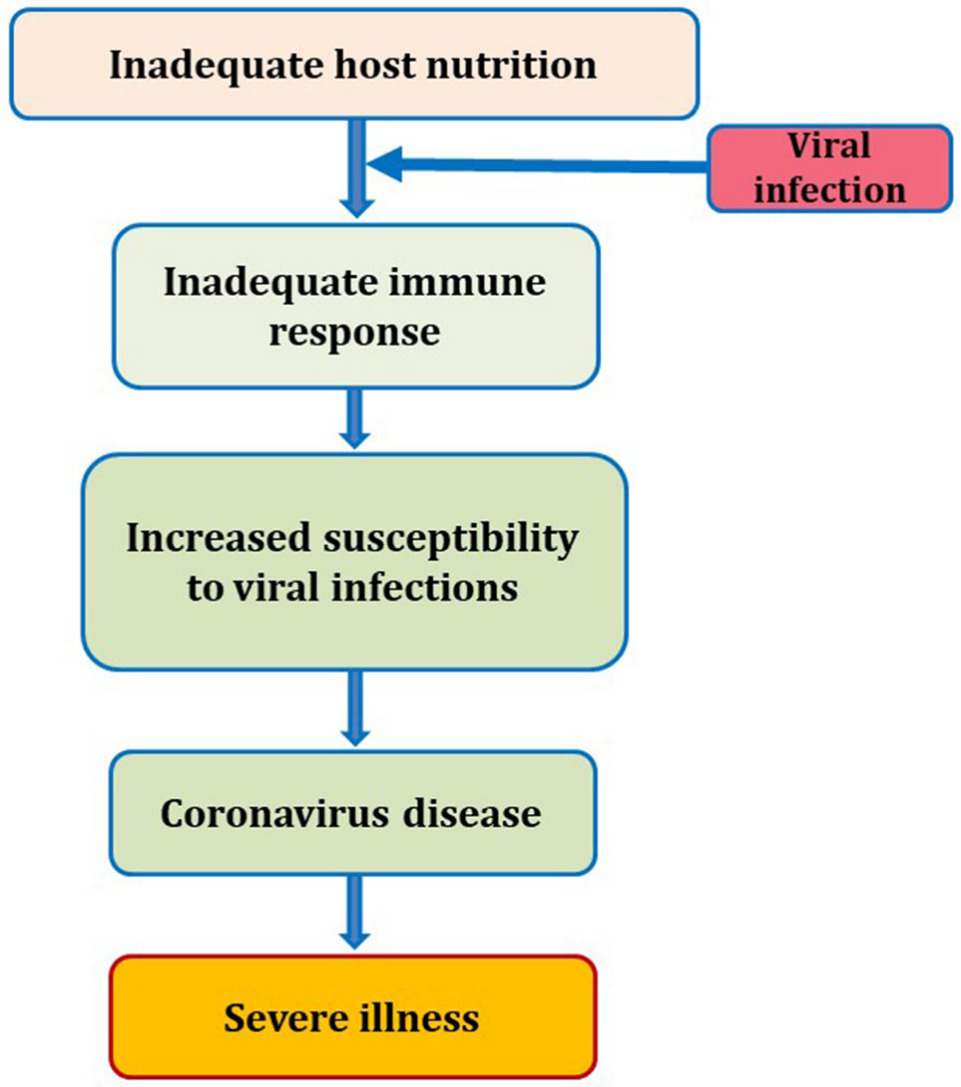
Schematic representation of role of nutrition in immunity against viral infections

In order the curtail the spread of the SARS-CoV-2, any countries have implemented several measures like quarantine, wearing mask, social distancing and temporary closure of food-related companies and markets to avoid spread of the corona virus disease 2019 (COVID-19). During this period, individuals feel world-weariness and associated with consumption of higher quantities of macronutrients such as carbohydrates, fats and proteins [3]. The increased consumption of macronutrients could also be accompanied by deficiency of micronutrients. This imbalanced dietary habits are commonly associated with the impaired immune responses which leads to severe illnesses (Fig. 1), affecting both the innate and adaptive immunity, and makes people more susceptible to viral and other infections [4].

The maintenance of an adequate micronutrient balance might strengthen the host immune response and to be protective of viral infections [5]. Essential micronutrients such as selenium (Se), zinc (Zn), copper (Cu), manganese (Mn), etc. have immunomodulatory functions and thus influence the susceptibility to the course and the outcome of a variety of viral infections [6]. The trace elements, coronavirus and immune system interactions highlighted the importance of trace elements in nutrition of host to optimize immune response to infections, and in preventing viral mutations which could increase the viral pathogenicity. Available electronic databases including Pubmed, Google and Scopus have been comprehensively searched for articles published with full text available and with the key words “Trace elements”, “COVID-19”, “Viral Infections” and “Immune Response” (i.e. separately Zn, Se, Fe, Cu, Mn, Mo, Cr, Li, Ni, Co) appearing in the title and abstract. On the basis of available articles, we have explored the role of trace elements in viral infections with special reference to COVID-19 and their interactions with the immune system.

## Dietary Importance of Trace Elements

**Fig. 2:**
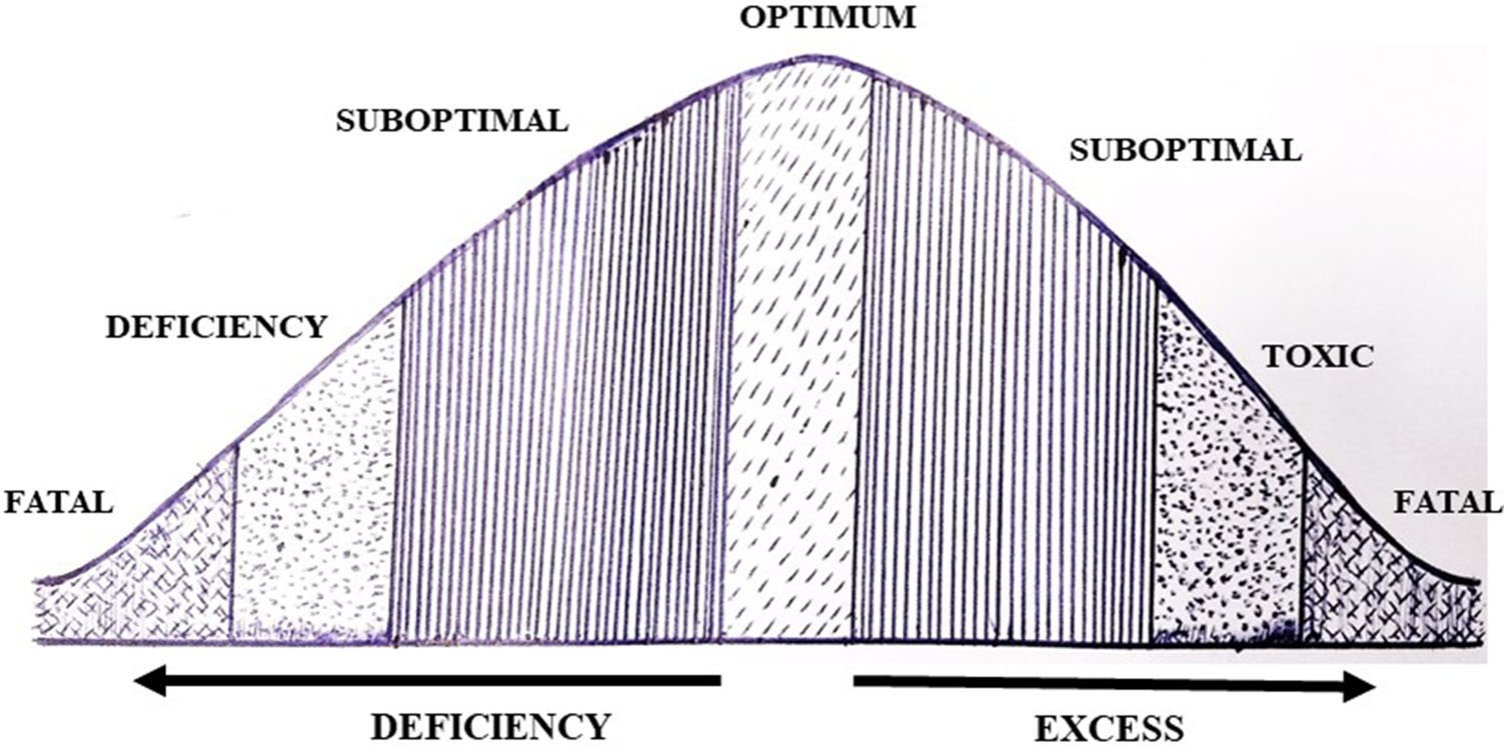
Potential adverse effects of trace elements in deficiency or overdose

Regular intake of trace elements gives immense significance to maintain general health. Recommended dietary allowance of various trace elements gives a clear structure for regular intake as a balanced diet to lead a healthy life [7] (Table 1). Intake of appropriate quantity is essential for performing the vital functions. Deficiency of trace elements results in sub-optimal functions, malnutrition, deficiency related diseases, while excess intake cause toxicity, which leads to fatal outcome (Fig. 2). Nutritional deficiency of trace elements are associated with impaired immunocompetence and increased susceptibility to infections [8].

**Table 1:** Recommended dietary allowances of essential trace elements to promote immune functions

S. no	Trace elements	Supplementation quantity for adult male/day	Supplementation quantity for adult female/day
1	Zinc	11 mg	8 mg
2	Selenium	55 μg	55 μg
3	Iron	8 mg	18 mg
4	Copper	900 µg	900 µg
5	Magnesium	400 mg	310 mg

There are eleven trace elements such as zinc, selenium, iron, copper, manganese, molybdenum, cobalt, iodide, fluoride, chromium and nickel. Evidences suggest that, vanadium, silicon and boron possess beneficial bioactivity in reasonable amounts [9]. Trace elements have proven to control the important cellular processes by binding to molecules on the receptor site of cell membrane or by alternating the structure of membrane to prevent entry of specific molecules into the cell [10]. Trace elements mediate vital biochemical functions by acting as cofactors for many enzymes such as glutathione peroxidise (GPx), superoxide dismutase (SOD), RNA polymerase, as well as act as centres for stabilizing structures of many enzymes and receptor proteins like Toll-like receptor-4 and transcription factors likeNf-kB [11, 12].

## Immunity Against Coronavirus Infections

Coronaviruses are zoonotic viruses mainly responsible for respiratory illness. SARS-CoV-2 belongs to genus of β-coronavirus. Phylogenetic analysis of its whole genome confirms its similarity to SARS-CoV. Generally human immune system fights against viral infections through a variety of specific and non-specific mechanisms. Several immune defence mechanisms can eliminate viruses from the host. Antigen presenting cells like dendritic cells and macrophages phagocytose the antigens and break down into fragments with the help of lysosomes. These fragments are loaded onto major histocompatibility complexes class I or class II molecules, and are transported to the cell surface for antigen presentation. Toll-like receptors on T cells along with co-receptors binds to displayed antigen. Helper T-cells secretes cytokines, which activates cytotoxic T (T_C_) cells and B cells. T_C_ cells kills virus infected cells through cell-mediated immunity (CMI) and B cells secreted antibodies carry out humoral immunity [13]. Upon SARS-CoV-2 infection releases enormous quantities of proinflammatory cytokines and chemokines by immune effector cells termed as ‘cytokine storm’. The cytokine storm triggers a violent systemic inflammatory response, sepsis, multi-organ failure and finally death in severe cases. The immune evasion mechanism of coronavirus is mainly due to inhibition of Type-I interferon (IFN) production. Lack of type I IFN leads to defect in antibody production, effector T-cell response, expression of IFN stimulating genes and also decreased antigen presentation [14, 15].

## Trace Elements in Immune Response Against COVID-19 and Other Viral Infections

### Zinc

**Fig. 3:**
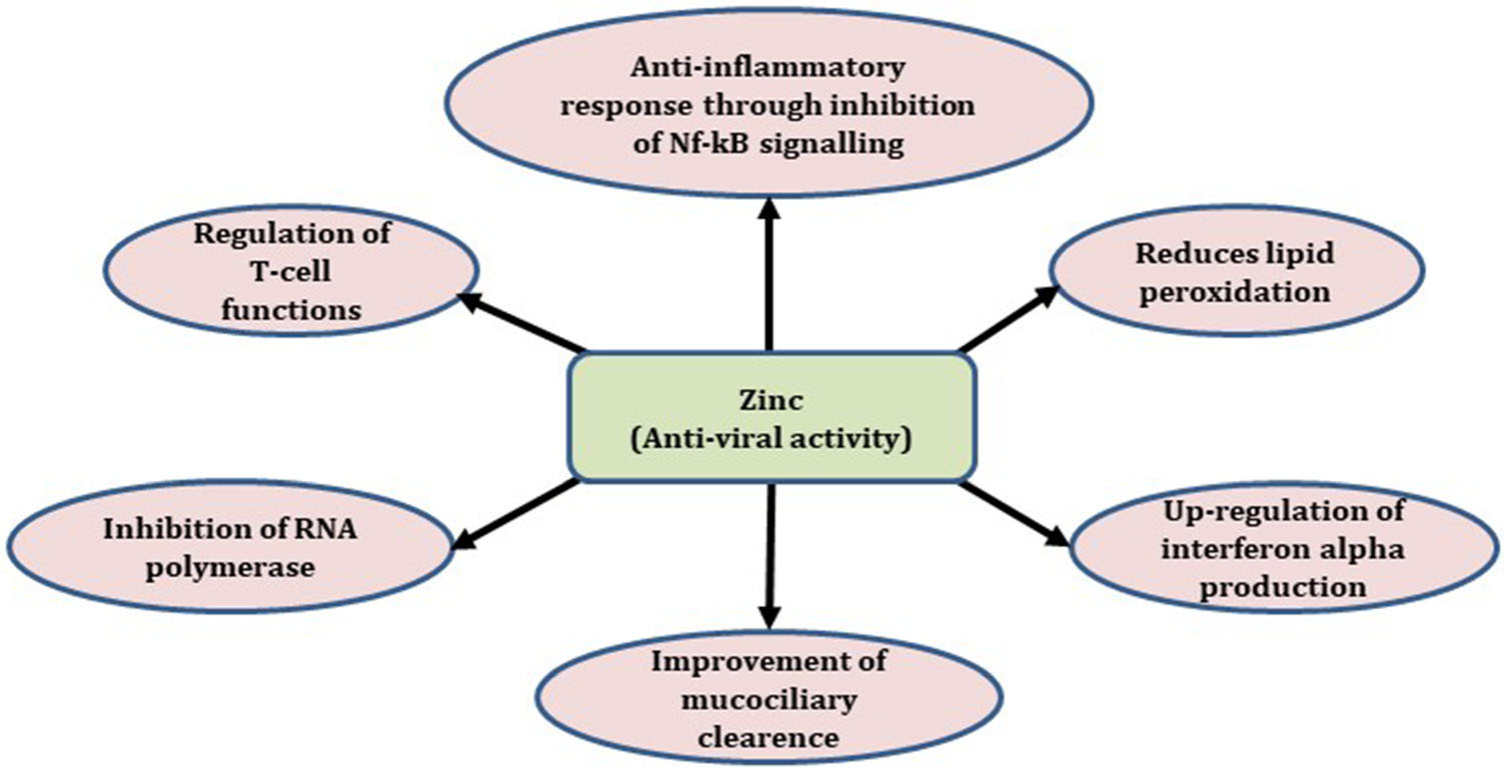
Biochemical and antiviral functions of zinc

Zinc (Zn) is an integral component of zinc finger motifs and transcription factors. Approximately, 20% of zinc is part of biomembrane proteins, 50% is present in cytosolic organelles and cytoplasm and 30% is found in nucleus [16]. An adequate concentration of Zn is essential for division, differentiation and maturation of T-lymphocytes, response of T-cells into mitogens, transcription of several immune regulatory genes in leukocytes. Severe Zn deficiency causes failure of both primary and secondary immune response. It is a component of Zn finger proteins, which act as transcription factors [17]. Zinc is essential for highly proliferating cells, especially in the immune system and influences both innate and acquired immune functions [18]. It is involved in the cytosolic defence against oxidative stress (SOD activity) and is an essential cofactor for thymulin, which modulates cytokine release and induces proliferation [19]. Zinc plays a pivotal role in central dogma and acts as cofactor in a number of enzymes, including RNA and DNA polymerases, ribonuclease and thymidine kinase. These enzymes are essential for immune cells growth, development, maturity and cell division (Fig. 3).

#### Role of Zinc Against Oxidative Stress and SARS-CoV Infection

Zinc is a divalent transition metal, key factor of antioxidant enzyme SOD. SOD converts superoxide (O_2_^**·**–^) to hydrogen peroxide (H_2_O_2_).

$${\text{O}}_{2}^{{ \cdot {-} }} + {\text{O}}_{2}^{{ \cdot {-} }} + 2{\text{H}}^{ + } \to {\text{H}}_{2} {\text{O}}_{2} + {\text{O}}_{2}$$

SARS-CoV-2 viral pathophysiological processes affect redox imbalance and oxidative stress. This phenomenon over produces the levels of reactive oxygen species (ROS) and deprived antioxidant defence mechanisms. A strong correlation between the oxidative stress markers and severity of viral diseases makes a crucial role for viral replication and its associated complications [20]. Several studies have suggested severe lung injury due to SARS-CoV triggers oxidative stress coupled exacerbation of proinflammatory response through NF-kB, transcription factor. Zinc accomplished versatile functions involved in antioxidant system, regulate inflammatory response and improved antiviral mechanisms and possess protective adjuvant therapy for COVID-19. Dietary Zn supplements provide beneficial impacts on health and amelioration of the SARS-CoV-2 pandemic situations. Trace elements play a non-specific activator of antioxidant metalloenzymes on viral infections. Antioxidants increase the number of T-cell subsets, increased interleukin-2 production, enhance lymphocyte response to mitogens, potentiated natural killer cell activity and increased response to influenza virus vaccine compared with placebo [21].

#### Role of Zinc in Immune System

Several studies showed that depending on the type of stimulus and concentration of Zn the immune system can either be stimulated or suppressed. Adequate Zn intake supports a T_H_1 response, and helps to maintain mucosal membrane integrity, and unbound zinc ions exert a direct antiviral effect on rhinovirus replication. Zinc supplementation increases cellular components of innate immunity (e.g. phagocytosis by macrophages and neutrophils, NK cell activity, generation of oxidative burst, DTH activity), antibody responses and the numbers of cytotoxic CD8^+^ T cells (T_H_1 response). Zinc deficiency results in lymphoid atrophy and decreased capacity to respond to many T-dependent antigens. High level of Zn is immunosuppressive, decreases the neutrophils and T-cell proliferation to mitogens and decrease antibody production. Thus, imbalance of Zn adversely affects immune functions [22]. Activity of thymic hormone decreases in deficiency of Zn, Cu and Se. Zinc deficiency of suboptimal levels in a mouse model reduced thymus size and depleted macrophages and lymphocytes in the spleen [23].

#### Role of Zinc Against Coronavirus Pathogenesis

**Fig. 4:**
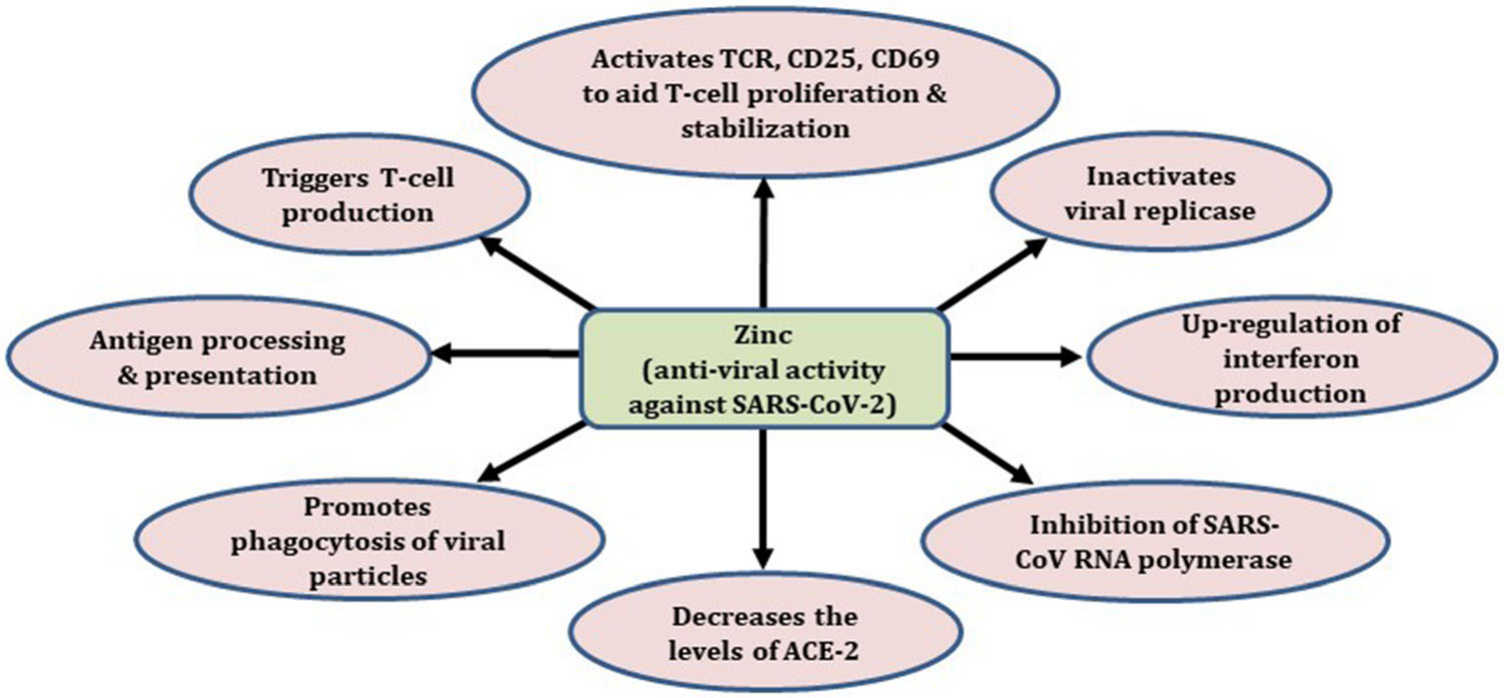
Various mechanisms of Zinc’s action against COVID-19

Zinc is a booster of immune system and plays a vital role in immunocompetence [24]. It is involved in development and maturation of immune cells and inflammatory response. Zinc acts as cofactor of cellular machineries of replicative and transcriptional enzyme systems such as DNA and RNA polymerases, respectively. In hepatitis C and rhinovirus, Zn showed antiviral activity by inhibiting RNA dependent RNA polymerase (RdRp) and other cellular cofactors [25]. Zinc inhibits the elongation phase of RNA synthesis in SARS-CoV, it might be due to effect on template binding. Proteolytic processing of replicase polyproteins and RdRp activity appears to be inhibited by zinc [26]. A distinctive feature of coronavirus replication is transcription of structural and accessory protein encoding genes in 5′- and 3′-coterminal nidus set of subgenomic (sg) mRNAs [27]. In cell free systems and infected cells the processing of coronavirus replicase polyproteins are inhibited by Zn through hindering proteolytic mechanisms [28]. Studies based on several RNA viruses like picornaviruses, respiratory syncytial virus and influenza virus illustrated that, addition of compounds like pyrithione, hinokitol and pyrrolidinedithiocarbamate increases cellular Zn concentration, which inhibits replication [29]. The antiviral activity of chloroquine may be, attributed to its action as a Zn ionophore (Fig. 4).

Zinc is necessary for apoptosis. Transcription of genes belongs to lymphoid and myeloid origins [30]. Severe Zn deficiency individuals have decreased immune function. Supplementation of zinc along with chloroquine (CQ) or its metabolite hydroxychloroquine (HCQ) is more effective in reducing covid-19 morbidity and mortality than CQ and HCQ monotherapy [31]. Hydroxychloroquine inhibits antigen processing and presentation and stops the release of virus into the cytoplasm by decreases the acidity in endosomes [32]. Sattar et al. published a case report stated that intake of zinc along with azithromycin and chloroquine fastens the recovery rate in COVID-19 patients [33]. Studies carried in Vero-E6 cells have been reported that, zinc inhibits SARS-CoV RdRp through template binding and elongation. Synergistic actions of zinc and quercetin inhibits transcription of RdRp during transcription of SARS-CoV-1 genome [34]. The viral entry and reproductive cycle of SARS-CoV-2 was almost similar to SARS-CoV, whereas angiotensin-converting enzyme 2 (ACE-2) acts as receptors for SARS-CoV-2 entry into type-IIpneumocytes [35]. Modulation of ACE-2 receptor levels by Zn treatment was considered as potential therapeutic strategy in COVID-19 treatment [36]. It was elucidated that exposure of 100 μM concentration of Zn reduces recombinant human ACE-2 activity in rat lungs [37]. Zinc supplementation boosters the innate immunity through increase the interferon-alpha production by leucocytes [38]. Although the concentration is near to physiological level the modulating effect on SARS-CoV2-ACE-2 interaction seems to be only hypothetical. In SARS-CoV infection ciliary epithelia of respiratory mucosal membrane were severely damaged which leads to loss of mucous clearance, lower respiratory tract infections and pneumonia [39]. In animal model, treatment with zinc increases the length of cilia, mucous clearance, frequency of ciliary beat and integrity of ciliary epithelium [40–42].

### Selenium

Selenium (Se) is essential for protein folding, calcium flux, activation, proliferation and differentiation of immune cells [43]. Selenium function in the form of selenoproteins/selenoenzymes like thioredoxin reductase (TxR) and GPx, plays a crucial role in redox regulations and antioxidant functions [44]. TrxR maintains the cellular pool of reduced thioredoxin. Reduced Trx is involved in regulation of DNA biosynthesis, activation of transcription factors and gene expressions in cell proliferation, apoptosis, migration and inflammatory pathways [45]. GPx is a cytosolic enzyme that requires reduced glutathione and hydrogen/lipid peroxides as substrates and converted into oxidized glutathione and water/oxygen as by products. Se promotes GPx and phospholipid levels in immune cells.

**Fig. 5:**
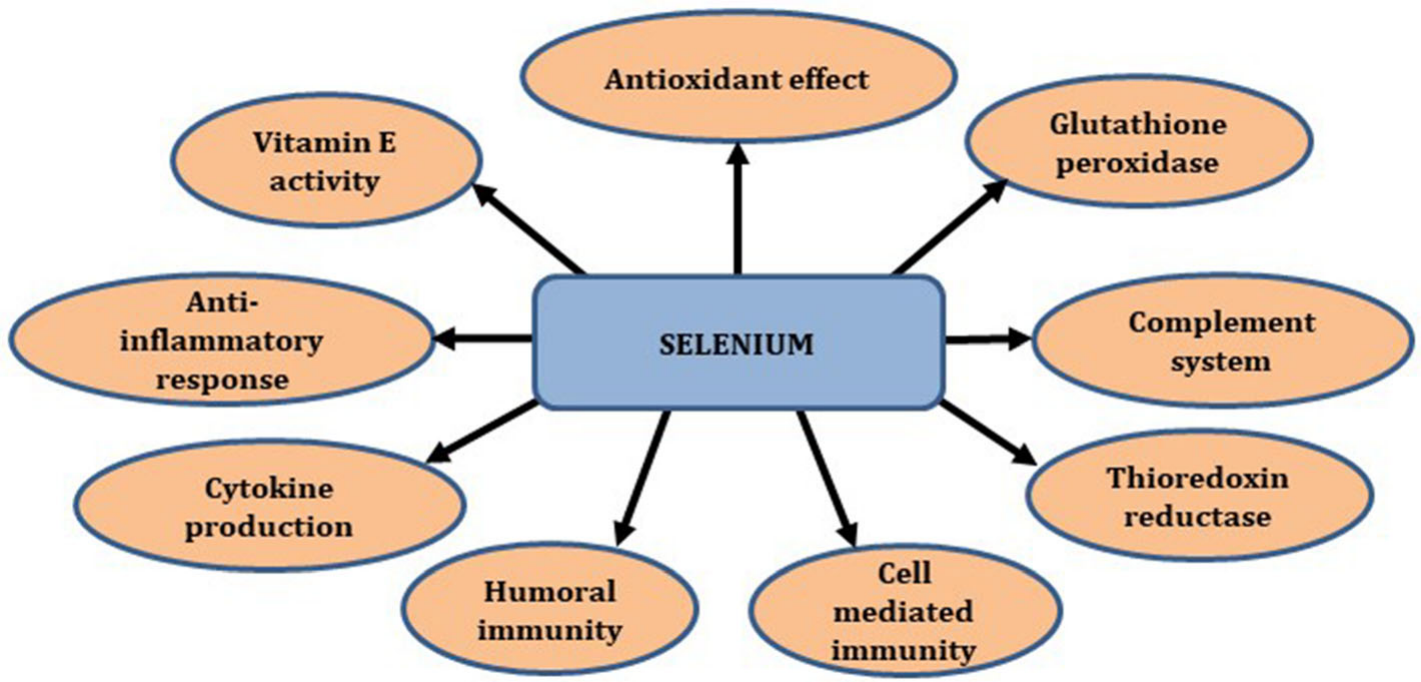
Various mechanisms of biochemical and antiviral activities of selenium

Selenium optimizes both innate and acquired immunity (Fig. 5). Low-dose supplementation of Se reduces humoral immunity against respiratory tract infections [46]. Selenium increases CMI through proliferation of T cells, increased IFN-γ and cytokines production. Selenium also promotes NK cell mediated immunity through perforin-granzyme pathways [47]. Selenium deficiency has shown to reduce the immune system and favours pathogenicity of coronaviruses. It inhibits virus replication in the host cells. The virulence property of SARS-CoV is mainly depends on its penetration property into pneumocytes [48]. Sodium selenite stops the viral infectivity by preventing the entry of coronaviruses into healthy host cells [49]. Antiviral mechanism of selenite is exhibited through oxidize thiol groups in viral proteins and renders penetration into host cell membrane [50]. One study demonstrated that Se deficiency in Hubei province increased the virulence of SARS-CoV-2 pathogenicity [51]. Decreased serum Se concentration causes accumulation of mutations in the genome of HIV, influenza A and SARS coronavirus, indicating the changes in the virulence is associated genetic structures [52]. Several studies demonstrated in animal model showed selenium deficiency increases mutation of viral genome which leads to increased pathogenicity and mortality [48, 53].

Complement system is the part non-specific immune system where C1qA plays an important role. Influenza virus protein M1 interacts with C1qA tightly, thereby escaping from the host immune systems [54]. Coronavirus has error-prone polymerase so that RNA can easily undergo several mutations and implicates the genetic diversity [55]. Selenium insufficiency causes oxidative damage to viral RNA and consequently increases the mutation rate. Increased mutation in viral genome favours the generation of pathogenic aggressive new strains. Selenium favours immuno-modulatory effects in many viral infections along with zinc. Adequate intake of nutrition showed better resilience and reduces the pathological conditions from COVID-19 infection [48]. In selenium deficient mice, several cytokines such as interleukin (IL)-4, IL-5, IL-10 and IL-13 were elevated, whereas IL-2 and IFN-γ were decreased. This modification might changes the pattern of T-helper cell function which fetches increased virulence property of coronaviruses [40]. Selenium consort with vitamin E, and prevent free radicals generation by assisting several types of enzymes. It prevents oxidative damage of immune cells and tissues. This synergistic effect induces immunity to a live bivalent infectious bronchitis coronavirus vaccine in chickens [56]. These observations suggested that intake of appropriate dose of Se may be effective therapeutic regimen for the treatment of SARS-CoV-2 infection.

### Iron

Iron is vital for electron transport chain reaction. Oxygen binds to iron in hemoglobin and transported to different tissues. Differentiation of cells and cellular growth is also regulated by iron. Enzymes that generate peroxide and nitric oxide also have iron as structural component and cofactor [57]. Cytokine production and its mechanism of action required iron for regulation. Phosphorylation requires protein kinase C activation which is an important signalling molecule for immune cell proliferation that requires iron for activation [58]. Either iron deficiency or overload had unfavourable functional cost to the immune system. Continued existence and replication of contagious viruses and microorganisms have necessity of iron and other micronutrients. Only optimum concentration is required for the proper functioning of immune system against infectious agents. In iron deficient stage there is slight reduction in rosette forming T cells and impaired lymphocyte proliferation retort to mitogens and antigens [24].

Iron distribution has to be tightly controlled to decrease the admittance of potentially harmful microorganism to this trace element. Deficiency of iron leads to change in T lymphocyte numbers, particularly of the CD_4_^+^ T_H_1 subpopulation [59]. Iron deficiency anemia in children is associated with the impaired maturation of T_H_ cells and is revived by iron supplementation [60]. In comparison with T_H_1 lymphocytes, T_H_2 lymphocyte has larger and less labile iron stores. Thus effect of iron deficiency on T_H_1 lymphocytes is more as compared to T_H_2 cells. Iron deficiency leads to impairment of proliferation of lymphocytes are due to either reduced translocation or activation of protein kinase C [61].

Housekeeping processes like energy production as cytochromes a, b and c, NADH and succinate dehydrogenase require metalloproteins whose structure and functioning depends upon iron. Microbiocidal factor like myeloproxidase dependent generation of hypochlorous acid require haem iron [61]. Atrophy of thymus occurs in iron deficiency, not because of increase in programmed cell death [62]. Iron deficiency also leads to decrease in circulating T lymphocytes in blood. T-helper and T-suppressor cells are very receptive to low iron levels.

Coronavirus replication requires sufficient amount of intracellular iron levels, whereas iron deficiency interferes in viral replication by impeding viral transcription, translation, assembly and exocytosis. Coronavirus enters the host cells by fusing its envelop with the host membrane and dismantle and release viral genome and nucleocapsid. Genomic RNA and structural proteins yield after transcription and translation. Coronavirus assembled in endoplasmic reticulum and Golgi apparatus after further processing and forms new progeny [63]. Finally, exocytosis of virion containing vesicles occurs. Iron containing enzymes and ATP is required during this process. Therefore, adequate amount of iron is required for coronavirus replication and iron deficiency make this process unfavourable [64–66].

### Copper

Copper (Cu) plays a vital role in the energy production through electron transport chain (ETC). Enzymes required copper for ETC are cytochrome C reductase and cytochrome oxidase. Certain enzymes like SOD require copper for its regulation and catalytic functions as enzymic antioxidant. Along with Cu, Zn is also necessary for the action of SOD (Cu, Zn-SOD), which can quench ROS. Copper plays a vital role by converting superoxide anions to oxygen and H_2_O_2_. Its deficiency can be diminished by dietary intake. It is also required for iron metabolism [67]. Copper in both excess and deficient condition alter the immune response. It plays a crucial role in the development, maturation and proper functioning of immune system. Copper deficiency causes decreased antibody forming cell response and increased susceptibility to a variety of viral infections [68]. Decreased number of circulating neutrophils known as ‘neutropenia’ is seen in Cu deficiency [69]. Severe Cu deficient rats exhibited reduced cellular Cu status, increased ROS and fungicidal activity on *Candida albicans* of peritoneal macrophages. Effectiveness of the acquired immune response is decreased in Cu deficiency. Copper deficient animals showed a decrease antibody production from spleen B cells [70]. Immunoglobulins and phagocytosis are comparatively low in Cu-deficient children. There is significant improvement in phagocytic index after Cu supplementation in those children. Proliferation of peripheral blood mononuclear cells was significantly decreased due to Cu deficiency [71].

Copper has the persuasive capacity to reduce the effect of infectious viruses such as bronchitis virus, poliovirus, and human immunodeficiency virus type-1 (HIV-1). Copper shows its neutralizing effect on some enveloped and non-enveloped single and double-stranded DNA and RNA viruses. Peroxide and Cu ion mixture showed synergistic effect of killing viruses than glutaraldehyde in herpes simplex viruses (HSV) [72]. Exposure of human coronavirus 229E to copper regulates its genes, its DNA and also virus morphology, including dissolution of envelope and thinning out of surface spikes [73]. Evidence suggested that, the n-COV showed high sensitivity on Cu surface and inactivated [74]. Cu^2+^ was shown to block papain-like protease-2, a protein that SARS-CoV-1 requires for replication in a cell-based study [75, 76].

### Lithium

Lithium (Li) exhibits anti-viral effect against coronavirus bronchitis virus [73]. It inhibits replication and cellular entry of coronavirus in Vero cells and suppresses transcription, translation of viral proteins in a dose dependent manner [77]. Lithium possess anti-inflammmatory effects by inhibiting COX-2 expression, IL-1β, TNF-α and increases the levels of IL-2, and IL-10. This activity of Li gives a clear figure to treat COVID-19 [78].

### Nickel

Nickel (Ni) is an essential micronutrient, which is found in cells and tissues at reliable levels, and is also associated with DNA and RNA in suggested quantity of physiological significance. It is essential for modulation of immune system. Administration of low dose of nickel chloride causes immunotoxic effect [79]. Several studies have proven that Ni increases spleen B and T cell activities and decreases NK cells activities [79]. It has been observed that human coronavirus is rapidly inactivated at room temperature (~ 21 °C) on alloy surfaces made up of copper and nickel, probably through generation of ROS [80] (Table 2).

**Table 2:** Status of trace elements in various viral diseases along with their reference intervals in healthy adult individuals

S. no	Trace elements	Reference ranges of healthy adult individuals	Trends of trace elements in viral diseases
1	Zinc	66–110 µg/dL [85]	Decreased [39]
2	Selenium	5.8–23.4 µg/dL [85]	Decreased [51]
3	Iron	12.5–26 mmol/L [86]	Decreased [59]
4	Copper	75–145 µg/dL [85]	Decreased [68]
5	Lithium	0.8–1.2 mmol/L [87]	Decreased [78]
6	Nickel	0.3–1.1 µg/L [88]	Decreased [80]
7	Manganese	4.7–18.3 µg/dL [85]	Decreased [89]
8	Chromium	2–3 nmol/L [90]	Decreased [91]
9	Fluoride	0.29–1.52 µmol/L [92]	Decreased [93]
10	Cobalt	1.9–7.6 nmol/L [90]	Decreased [94]
11	Iodine	40–80 µg/L [95]	Mechanism unclear [96]
12	Molybdenum	0.28–1.17 ng/mL [97]	Decreased [98]

## Estimation of Trace Elements in Whole Blood/Serum Samples

The levels of trace elements can be estimated from serum/whole blood samples of SARS-CoV-2 infected patients. The blood samples are collected by lithium heparin anticoagulant containing vacutainers. Inductively coupled plasma–mass spectrometry (ICP-MS) and inductively coupled plasma-optical emission spectrometry (ICP-OES) can be used for quantification of trace elements which are found in the μg/mL or ng/mL concentration ranges [81]. Other techniques includes ion selective electrodes (ISE), photometry, emission spectroscopy (ES), neutron activation analysis (NAA), anodic stripping voltammetry (ASV), atomic absorption spectrophotometry (AAS), high performance liquid chromatography (HPLC) and Total Reflection X-ray Fluorescence (TXRF) are used for quantification of trace elements. TXRF is a most proposed technique for quantification of trace elements. It is a cost effective, reliable method and can detect trace elements and their interactions in broad variety of biological samples. In testing body fluids, many sequential single element determination techniques are available. TXRF is a multi-trace element determination technique, time saving and requires minimal biological samples for analysis whereas, ICP-MS and ICP-OES are time consuming and requires lot of time for sample preparation. The main advantages of TXRF is picogram range of detection limit and more precise technique [82]. Trace element like copper levels can also be measured by functional tests, for example erythrocyte superoxide dismutase, serum ceruloplasmin and cytochrome c oxidase assays. Fluoride level is also measured by ion-specific potentiometry [83].

## Conclusions

Trace elements are essential for activation, development, differentiation and perform numerous functions of immune cells. Molecular mechanisms of trace elements such as zinc, selenium and copper are fairly elucidated against SARS-CoV-2 infections [49, 84]. Few studies also available on the role of nickel and lithium in COVID-19 and other viral infections. Many experimental studies (both in vivo and in vitro) are ongoing to elucidate the molecular mechanisms of trace elements in anti-viral immunity of SARS-CoV-2. Minor trace elements have anti-viral activity on certain viruses such as influenza virus, HSV, HIV and BVDV viruses have been already demonstrated. Trace elements supporting the function and development of human immune system might reduce the risk of COVID-19. All the above described nutritional importance of trace elements represents a healthy life style to be followed in normal time and quarantine period of COVID-19 infections. Intake of adequate sources of immune supportive micronutrients, proper time for eating along with positive approaches could be helpful for combating this COVID-19 pandemic situation.

### Future Perspectives

Micronutrients have equal potential as like macronutrients with regards to development and maturation of immune system. Deficiency of micronutrients is one of the major public health concerns in the world. So far, based on the studies revealed trace elements have the immunomodulatory effects at various aspects. But still molecular mechanisms of many trace elements related to innate and acquired immunity are under reviled.

## Abbreviations

ACE-2: Angiotensin converting enzyme-2
cGAS: Cyclic GMP-AMP synthase
CMI: Cell-mediated immunity
COVID-19: Coronavirus disease 2019
SARS-CoV-2: Severe acute respiratory syndrome coronavirus-2
Co: Cobalt
CQ: Chloroquine
Cr: Chromium
Cu: Copper
ETC: Electron transport chain
GPx: Glutathione peroxidase
HCQ: Hydroxychloroquine
HSV: Herpes simplex viruses
IL: Interleukin
Li: Lithium
MERS: Middle East respiratory syndrome
Mn: Manganese
Mo: Molybdenum
n-COV: Novel coronavirus
Ni: Nickel
ROS: Reactive oxygen species
SARS: Severe acute respiratory syndrome
Se: Selenium
SOD: Superoxide dismutase
Tc: Cytotoxic T-cells
TxR: Thioredoxin reductase
Zn: Zinc
